# Superimposed visceral leishmanial infection aggravates response to *Heligmosomoides polygyrus*

**DOI:** 10.1186/s13071-018-2987-1

**Published:** 2018-07-11

**Authors:** M. E. González-Sánchez, M. Cuquerella, J. M. Alunda

**Affiliations:** 1 Department of Animal Health, Faculty of Veterinary Medicine, Universidad Complutense de Madrid, 28040 Madrid, Spain; 2 Instituto de Investigación Hospital Doce de Octubre, Avda. Andalucía s/n, 28041 Madrid, Spain

**Keywords:** *Heligmosomoides polygyrus*, *Leishmania infantum*, Co-infections, IgG_1_, IgG_2a_, Lymphoproliferation, ELISA, BALB/c mice, Helminth

## Abstract

**Background:**

Polyparasitism is the rule in all animal species, including humans, and has an important role in pathogenicity, diagnosis and control measures. Among them, co-infections by gastrointestinal helminths and protists are very prevalent under natural conditions but experimental infections are relatively scarce. Thus, despite the frequent association of visceral *Leishmania* infections and intestinal helminth parasitism the experimental co-infection has not been addressed. *Heligmosomoides polygyrus*, an intestinal nematode of mice, is related to other helminths causing important pathologies and is a model species for immunological studies. Mice are valuable experimental model for visceral leishmaniasis.

**Methods:**

BALB/c mice infected with *H. polygyrus* (200 third-stage larvae, L3) were subsequently infected seven days later with *Leishmania infantum* (10^7^ promastigotes) with the aim of determining the effect of the overinfection on the host response to the primary infection with the helminth.

**Results:**

Overinfection with the protist did not affect the establishment rate of the nematode but induced a higher fecal egg output. Helminth burdens in co-infected animals were significant at the end of the experiment. Early unspecific immune suppression induced by the nematode in mesenteric lymph nodes was not switched by *L. infantum* infection. Co-infection elicited a higher serum antibody (IgG_1_) response against the helminth.

**Conclusions:**

Visceral leishmanial overinfection aggravated the early host response against primary infections with the intestinal helminth. This effect was evidenced by an increased longevity and higher production of non-protective antibodies.

## Background

Gastrointestinal nematodiases are a major problem worldwide, both for human and animal health. Helminth infections are present in all species of wild and domestic animals, with different management practices and climates. Soil-transmitted helminthiases (STHs) are among the most common and persistent parasitic infections worldwide and it has been estimated that over three billion people are infected with one or more helminth species [1]. In the veterinary arena these diseases have a strong impact on health status, productivity and therefore on the sustainability of animal production. Visceral leishmaniasis is the second most lethal parasitic disease for humans [2]. *Leishmania infantum* zoonotic infection is found in the Mediterranean and Brazil where millions of dogs are naturally infected.

Multiparasite infections are the rule and the coexistence of more than one species in a host has significant effects on their pathogenicity, clinical course and design of control systems [3, 4]. Information is relatively scarce and only recently has a growing interest on the study of multiparasitism been observed [5, 6].

*Heligmosomoides polygyrus* (Nematoda: Strongylida), a natural parasite of mice intestines, is a species taxonomically related to those causing processes of relevance in humans (e.g. *Necator*, *Ancylostoma*) and in animal health and production (e.g. *Ostertagia*, *Haemonchus*). Primary exposure to *H. polygyrus* provokes a predominant Th2 response [7] accompanied by regulatory T cell (Treg) activation, thus failing to achieve an effective expulsion of the worms and causing chronic infections in most mouse strains [8–10]. The lack of protective response in primary infections is related to the immunosuppressive abilities of the nematode since an effective response appears when the primary infection is cleared (i.e. with drug treatment) [11]. Immune interference between helminths and protozoans has been described and experimentally addressed in some combinations [12–16]. Cross-sectional studies have been carried out in human patients with cutaneous (caused by *Leishmania braziliensis*) [17] and visceral leishmaniasis [18] and helminth infections with conflicting results. However, the potential effect of a visceral *Leishmania* infection on previously infected hosts with intestinal helminths has apparently not been experimentally addressed. This lack of information is surprising, given the widespread distribution of helminths all over the world and the high prevalence of visceral leishmanial infections. Since *H. polygyrus* infections are a well established model of Th2 response in mice and *L. infantum* infection in mice elicits a mixed Th1/Th2 response [19–21], we administered an overinfection with *L. infantum* to BALB/c mice previously infected with *H. polygyrus*. Results obtained in this surrogate model showed that visceral leishmanial infection aggravated the primary response against the intestinal helminth.

## Methods

### Parasites

*Heligmosomoides polygyrus* larvae were provided by M. Grueiro (Faculty of Pharmacy, UCM, Madrid, Spain) and the isolate was maintained in our laboratory by passage in susceptible mice every 6 months. The infective third-stage larvae (L3) were obtained by incubation of fecal material on filter paper disks placed on a Petri dish with distilled water at 22 °C for 7 days. The isolate of *L. infantum* (M/CAN/ES/97/10.445 zymodeme MON-1) was supplied by M. Domínguez (ISCIII, Madrid, Spain) and has been maintained as promastigotes by passage in RPMI 1640 medium (Lonza Group, Basel, Switzerland) at 26 °C supplemented with heat inactivated fetal bovine serum (30 min, 56 °C) (Sera Laboratories International, Horsted Keynes, UK) and 100 U/ml penicillin + 100 μg/ml streptomycin (BioWhittaker, Verviers, Belgium).

### Mice, experimental design and follow-up

Female BALB/c mice (Harlan Laboratories Models SL, Barcelona, Spain) were housed in our facilities (No. ES280790000155) in polystyrene cages (4 animals per cage) at a controlled temperature of 22–25 °C with a 12 h light 12 h darkness cycle and received water and commercial rodent feed *ad libitum*. Mice were randomly allocated to four experimental groups of eight animals per group. G1 and G2 animals were infected with 200 L3 of *H. polygyrus* in 0.2 ml water, using a bucoesophagic catheter, on day 0 of the experiment. G1 and G3 animals were infected by intraperitoneal injection, on day 7 post-infection (pi), with 10^7^ stationary promastigotes of *L. infantum* in 0.1 ml PBS. G4 was the uninfected control group.

Individual blood samples were obtained on day -1, 6, 14, 21, 28 and 35 pi with *H. polygyrus* by puncture of submandibular vein. The blood volume obtained was 50 μl/ mouse/sample day except for the days when animals were euthanatized (14 and 35 pi) when 150 μl were taken. Blood was allowed to clot and the sera preserved at -20 °C until used. Samples taken at the end point of the experiment were employed for ELISA determinations. Coproscopical analyses were performed every 3 days from the 9th day pi onwards. Mice were individually isolated for 30 min, their feces collected and egg counts performed by a modified flotation technique. The results were expressed as eggs per gram of feces (epg). Cumulative epg output was estimated using the trapezoidal method to determine areas under the curve (AUC) of the animals and groups. Mice were euthanatized (CO_2_ inhalation - isoflurane) on days 14 and 35 pi, 4 animals per group at each time point. Intestines, spleens and mesenteric lymph nodes were removed and employed for further determinations. Individual intestines were opened and placed in physiological saline solution, kept overnight at 37 °C and the worms were recovered and counted. Spleens were extracted under sterile conditions and weighed individually. To assess *L. infantum* infection, a spleen sample was employed to prepare smears, stained with May Grünwald Giemsa and microscopically examined.

### Antigens

*Heligmosomoides polygyrus* soluble extract (ASE) was obtained from adult helminths. Nematodes were cleaned with cold PBS containing protease inhibitors (Roche, Mannheim, Germany), subjected to 8 freeze-thaw cycles (-80 °C to room temperature), homogenized in a glass-in-glass homogenizer and centrifuged at 3000× *g* for 30 min at 4 °C. Soluble leishmanial antigen (SLA) was obtained by 10 freeze-thaw cycles of *L. infantum* promastigotes followed by centrifugation and recovery of the supernatant. The concentration of protein was measured [22] and the antigen extracts (SLA, ASE) stored at -80 °C until use.

### Lymphoproliferation

Lymphocyte proliferation was determined using the tetrazolium salt (MTT) method [23] with some modifications. Mesenteric lymph nodes (MLN) and spleen were dissected individually and homogenized in medium RPMI 1640. The suspension was filtered (100 μm mesh), centrifuged at 300× *g*, 10 min, 4 °C and the pellet resuspended in medium with 10% fetal bovine serum, 100 U/ml of penicillin + 100 μg/ml streptomycin and 1% glutamine. Erythrocytes were lysed with red blood cells lysis buffer (0.15M NH_4_Cl + 10mM KHCO_3_ + 0.1M Na_2_EDTA, pH7.2). The viability was assessed with Trypan blue. Viable cells from MLN (2 × 10^6^ cells/ml) and spleen (5 × 10^6^ cells/ml) were seeded in 96-well flat bottomed plates (Corning, Corning, USA; 200 μl/well) and stimulated with Concanavalin A (GE Healthcare, Madrid, Spain; 5μg/ml), SLA and ASE (5 μg/ml for MLN; 10 μg/ml for spleen). The cells were incubated for 96 h at 37 °C in a humidified atmosphere of 5% CO_2_. Proliferation was determined by adding 50 μl MTT (5 mg/ml) (Sigma, St. Louis, USA) + 200 μl culture medium for 4 h at 37 °C. Absorbance was read at 570 nm. All determinations were performed in triplicate and negative controls were included. Results were expressed as stimulation index (SI): optical density (OD) of stimulated cell cultures/OD of unstimulated control wells. SI ≥ 1.5 were considered positive.

### Enzyme linked immunosorbent assay (ELISA)

Serum specific antibody response (IgG_1_ and IgG_2a_) was determined by ELISA. Optimal assay conditions were determined in a checkerboard manner. Briefly, microtitre plates (Nunc, Roskilde, Denmark) were coated with 5 μg/ml ASE or SLA. Anti-mouse IgG_1_-HRP was from Nordic Immunology (Eindhoven, Netherlands) and HRP anti-IgG_2a_ was from Southern Biotech (Birmingham, USA). Mice sera were diluted 1/800 and conjugate 1/2500 to determine IgG_1_ against ASE; 1/50 sera dilution and 1/1000 conjugate dilution to estimate anti-SLA response. IgG_2a_ against ASE employed sera diluted 1/400 and 1/2000 dilution conjugate. Anti-*Leishmania* IgG_2a_ was determined with 1/25 sera dilution and 1/2000 conjugate.

### Statistical analysis

Intergroup differences were analyzed by t-test (adult helminthes, epg) or 2-way ANOVA (epg, spleen weight, lymphoproliferative response, ELISA results) followed by Tukey’s HSD test. Exact *P*-values, degrees of freedom (*df*) and analysis carried out are given using the APA format. The level of significance was set at *P* < 0.05. Figures were prepared with Graphpad Prysm 5.

## Results

### Overinfection with *L. infantum* provokes higher fecal egg output of *H. polygyrus-*infected mice

**Fig. 1:**
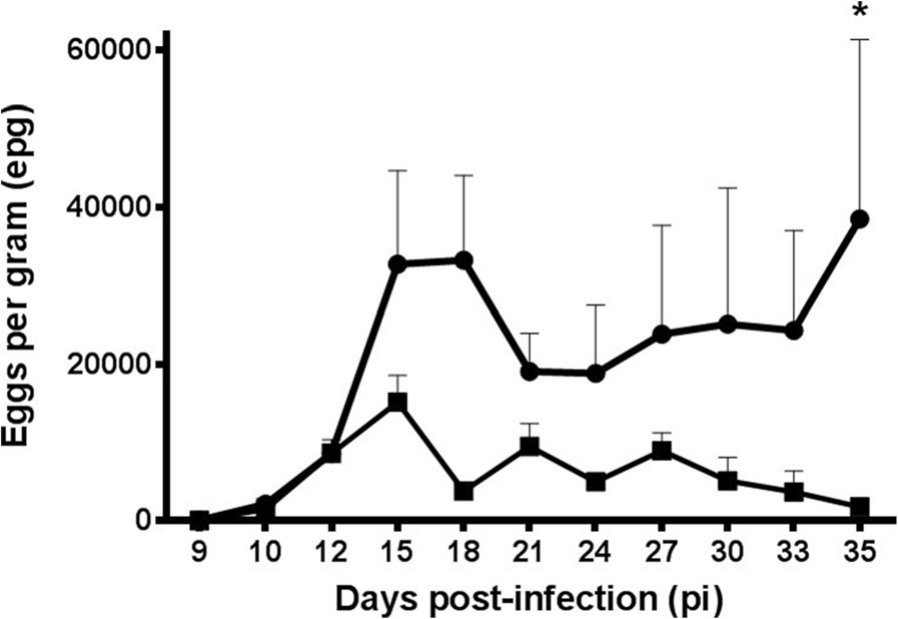
*Heligmosomoides polygyrus* egg excretion (eggs per gram, epg; mean ± SE) in infected mice with (G1, circles) or without superimposed *Leishmania infantum* infection (G2, squares).*P < 0.05

Animal groups infected with *H. polygyrus* (G1 and G2) started eggs excretion on day 10 pi; however, the epg pattern greatly varied depending on the infections received (Fig. 1). There was a high intragroup individual variability within a sample day and along the experiment. Thus, cumulative egg excretion, estimated by AUC from day 15 to the end of the experiment, showed that mice infected only with *H. polygyrus* shed *c.*25% of the AUC value of co-infected mice (G1: *H. polygyrus* + *L. infantum*) although the difference was not significant. There was a different pattern of epg values between animal groups and egg excretion (AUC) of co-infected mice was significantly higher between days 15 and 21 pi (*t*_(6)_ = 2.342, *P* = 0.0288). No eggs were found in the mice infected only with *L. infantum* (G3) and the uninfected control group.

### *Leishmania infantum* overinfection does not affect establishment of *H. polygyrus* although it increases helminth survival

**Fig. 2:**
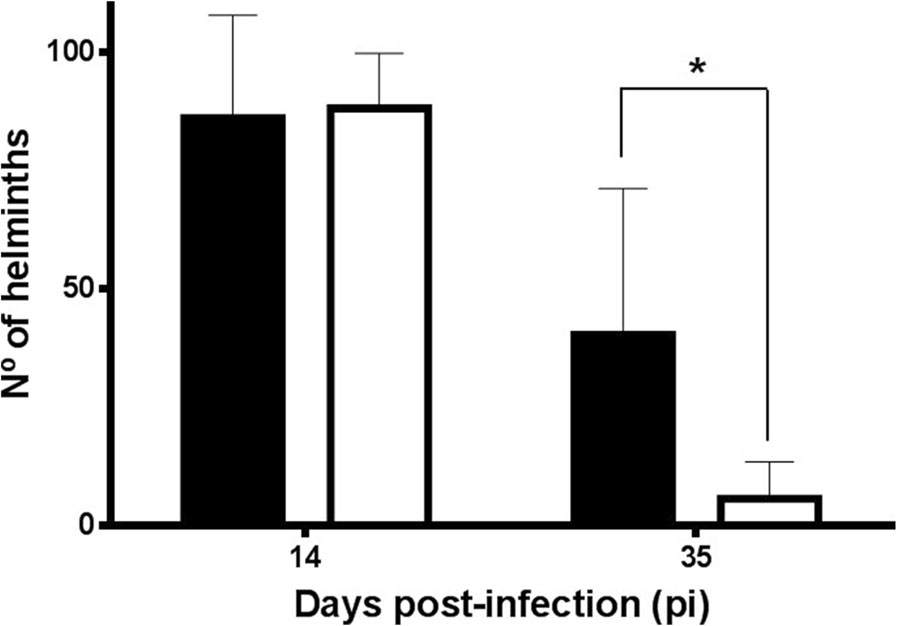
Intestinal helminth burden (mean ± SE) of *H. polygyrus*-infected mice with (G1) or without (G2) *L. infantum* overinfection. Solid bars, G1; empty bars, G2. *P < 0.05

On day 14 pi, estimated total worm burden was comparable in both mice groups (G1: 86.25 and G2: 88.25 helminths) and no differences were found in sex ratio of helminths (Fig. 2). Conversely, 35 days pi parasite burdens were significantly different depending on the presence of concurrent *L. infantum* infection. In a similar way to that found in epg patterns, mice co-infected (G1) hosted higher worm burdens than those observed in animals subjected only to *H. polygyrus* infection (*t*_(6)_= 2.188, *P* = 0.0357). The sex ratio of the adult population at the end-point varied according to the life-cycle of the helminth with a predominant female population (*c.*70%) in both animal groups.

### Spleen enlargement of co-infected mice relates to *H. polygyrus* infection

**Fig. 3:**
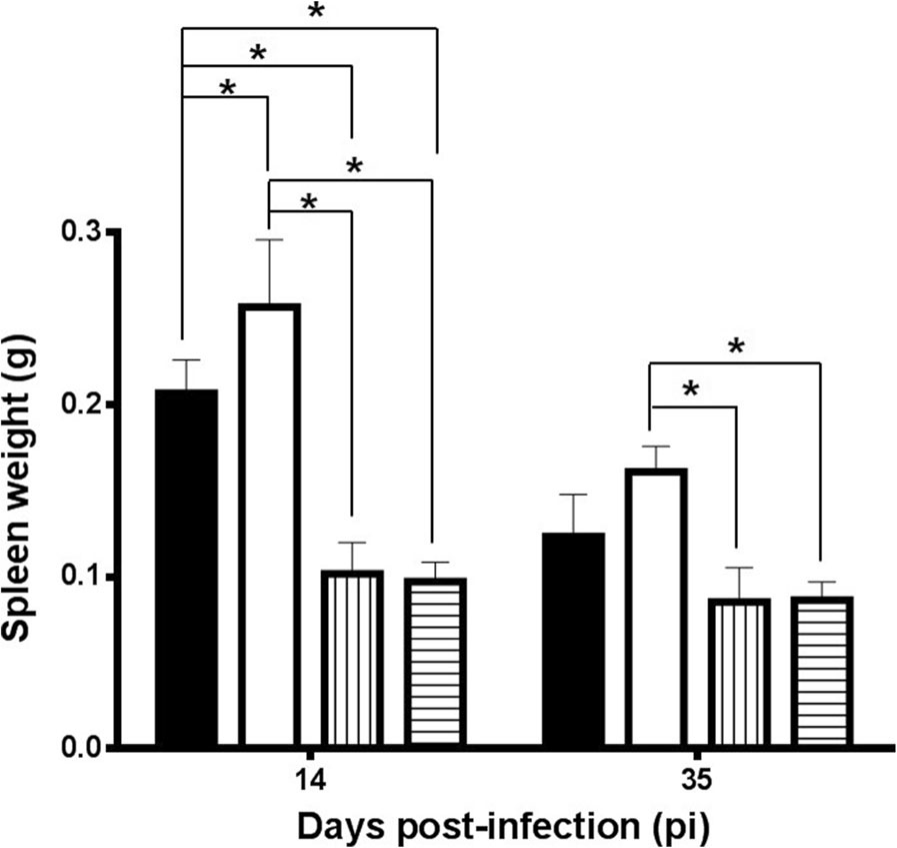
Spleen weight (mean ± SD) from mice at early patency (14 days pi) and the end of the experiment (35 days pi). Solid bars, G1; empty bars, G2; vertical striped bars, G3; horizontal striped bars, G4. *P < 0.05

There were significant differences among groups depending on the infection schedule (*F*_(3, 24)_ = 56.85, *P* < 0.0001), time of sampling (*F*_(1, 24)_ = 46.20, *P* < 0.0001) and the interaction (*F*_(3, 24)_ = 8.482, *P* = 0.0005) (Fig. 3). Tukey’s analysis showed that infection with *H. polygyrus* induced a significant splenomegaly of the animals since the spleen weights found in the group infected only with the nematode (G2) were significantly higher than those found in the group infected with *L. infantum* (G3) and the uninfected control group (G4) in the two samplings carried out (days 14 and 35 pi). This finding was particularly clear on day 14 of the experiment when the mean spleen weight found in the mice infected only with *H. polygyrus* was 2.64 times higher than that found in the control group (mean ± SD; G2: 0.2565 ± 0.0390; G4: 0.0973 ± 0.0112). In our experimental conditions *L. infantum* infection did not elicit any significant increase in spleen size. Spleen enlargement was found in all groups infected with the nematode. However, overinfection of *H. polygyrus*-infected mice with *L. infantum* (G1) induced a slight reduction of spleen weight at the beginning of the patency (day 14) (G1: 0.2066 ± 0.0193) when compared to mice only subjected to the helminth infection (G2: 0.2565 ± 0.0390).

### Early unspecific immune suppression elicited by *H. polygyrus* is not switched by *L. infantum* overinfection

**Fig. 4:**
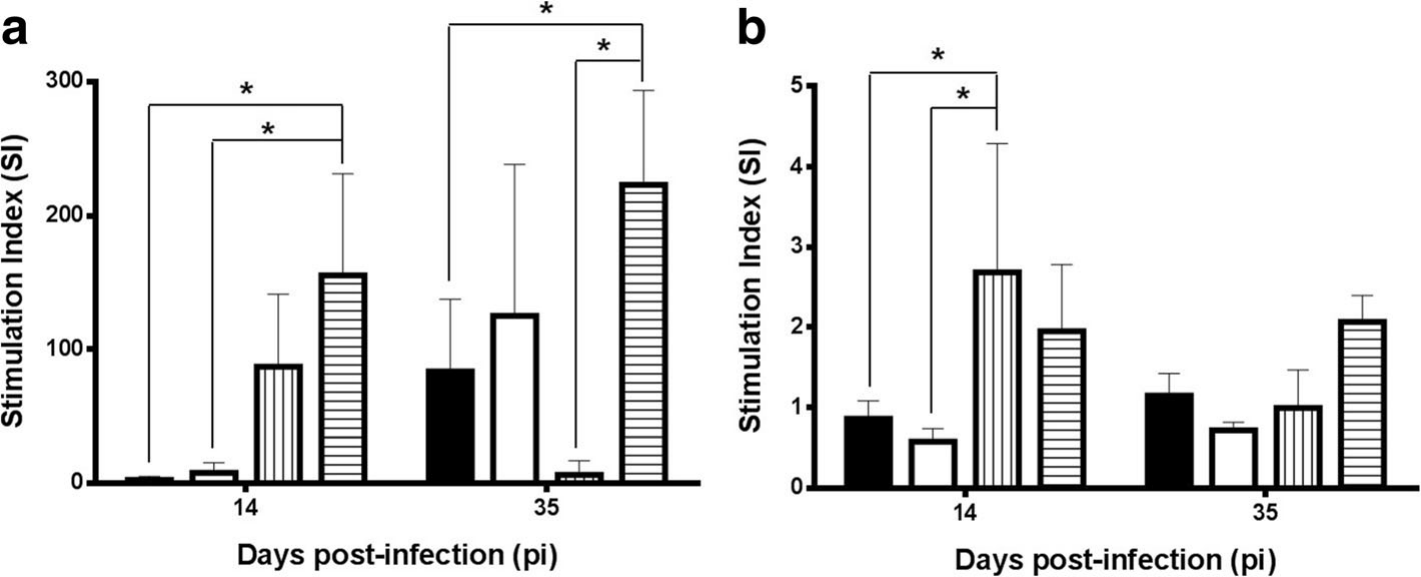
Lymphoproliferative response (mean stimulation index, SI ± SD) in the presence of Con A in mesenteric lymph nodes (**a**) and spleen (**b**) on days 14 and 35 pi. Solid bars, G1; empty bars, G2; vertically striped bars, G3; horizontally striped bars, G4. *P < 0.05

Infections by both *L. infantum* and *H. polygyrus* elicited unspecific immunosuppression as assessed by the lower lymphoproliferation found in mesenteric lymph nodes (MLN) (Fig. 4a; *F*_(3, 24)_ = 4.096, *P* = 0.018) and spleen (Fig. 4b; *F*_(3, 24)_ = 3.753, *P* = 0.024) at both time-points when cells were exposed to ConA. However there were infection- and time-course-related variations. Thus, in MLN an early immune suppression was more evident in the presence of *H. polygyrus* infection (G1: 2.590 ± 2.550; G2: 7.854 ± 7.554) compared to the uninfected mice (G4: 155.273 ± 76.266). A partial restoration was observed after 35 days of infection and at this time-point mice infected only with *H. polygyrus* (G2) were not significantly different to uninfected control animals. This early immune suppression was also observed with spleen cells. In the case of *L. infantum*, infection immune suppression followed a different pattern. Thus, on day 35 only mice infected with this parasite (G3: 6.264 ± 10.638) and to a lesser extent the co-infected group (G1: 83.390 ± 54.134) had significantly lower lymphoproliferative values than the control animals (233.145 ± 70.318). These results were particularly clear with MLN cells although the pattern was also found with spleen cells.

**Fig. 5:**
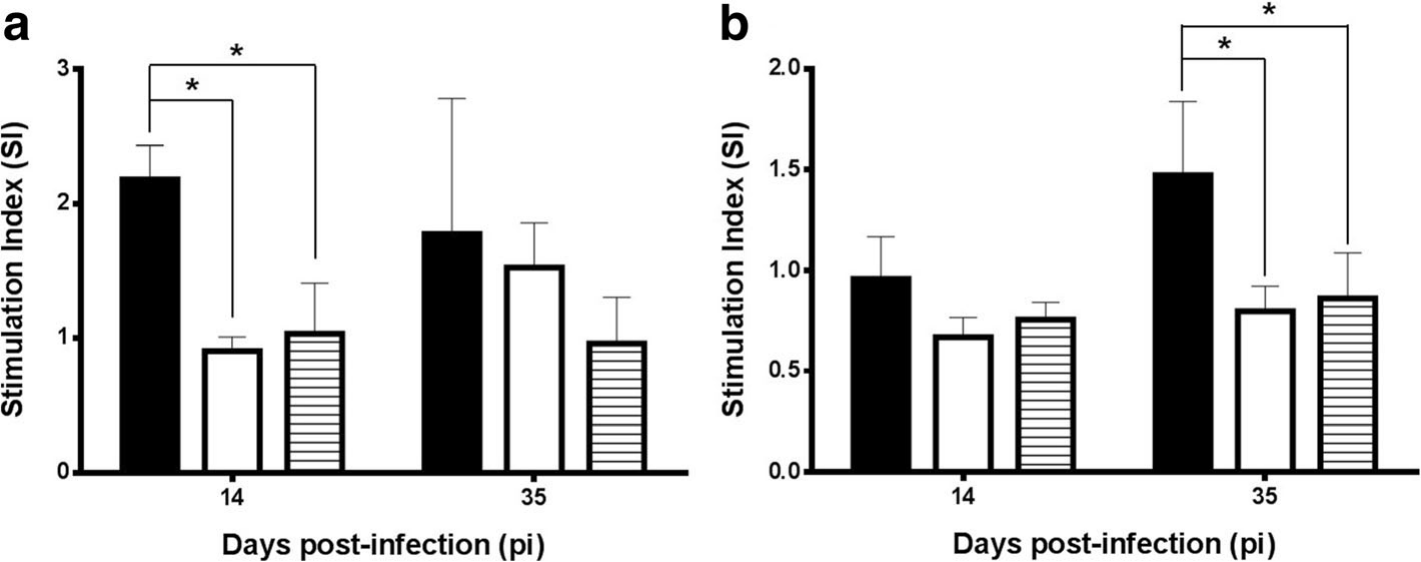
Specific lymphoproliferative response (mean stimulation index, SI ± SD) to adult soluble extract of *H. polygyrus* in mesenteric lymph nodes (**a**) and spleen (**b**). Solid bars, G1; empty bars, G2; horizontally striped bars, G4. *P < 0.05

Specific lymphoproliferation in MLN (Fig. 5a) and spleen (Fig. 5b), in the presence of *H. polygyrus* soluble antigen was low in all groups. Despite the helminth-induced unspecific suppression, the most reactive animals in MLN were those co-infected with *L. infantum* (*F*_(2, 18)_ = 8.762, *P* = 0.002] with significant differences on day 14 (2.181 ± 0.251) to both the control group (1.032 ± 0.376) and the animals with monospecific helminth infection (0.904 ± 0.104). This pattern was also observed after 35 days although differences were not significant. In the spleen (Fig. 5b) co-infected mice (G1) also displayed a higher proliferative response in the last sampling (1.474 ± 0.365). Lymphoproliferative response against leishmanial antigen was low and not related to the infection schedule of the animal groups (not shown).

### Overinfection with *L. infantum* increased the antibody response to *H. polygyrus*

**Fig. 6:**
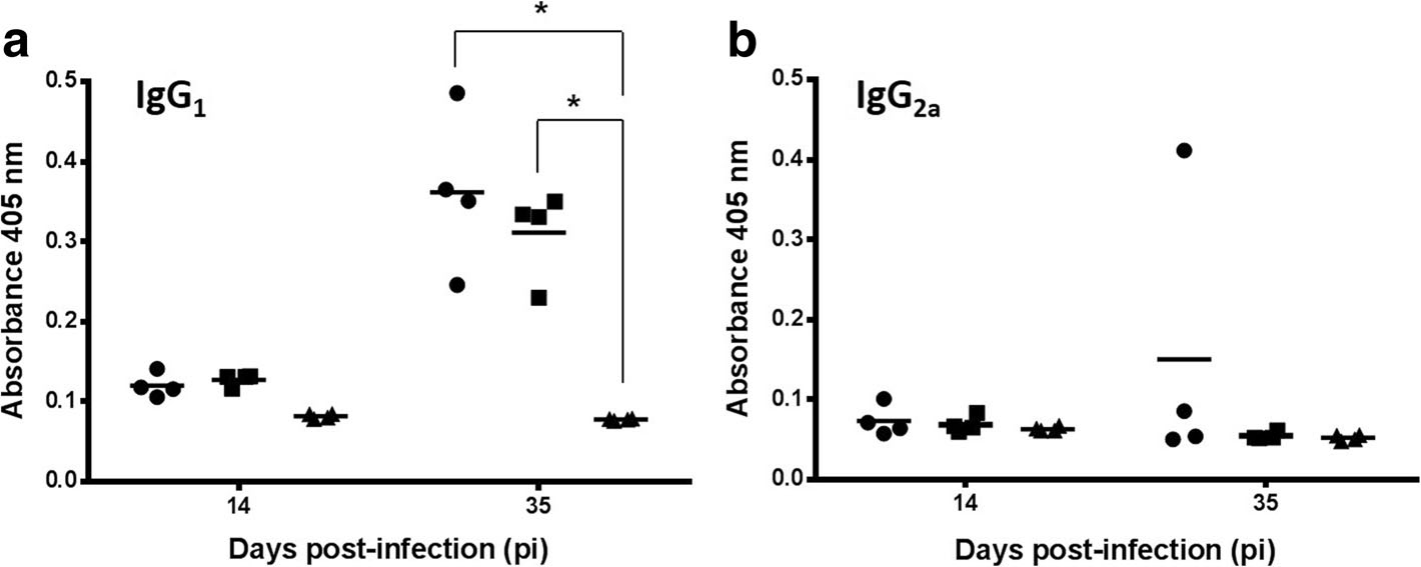
Individual serum IgG_1_ (**a**) and IgG_2a_ (**b**) levels of experimental mice on days 14 and 35 pi against *H. polygyrus* adult soluble extract (ASE) estimated by ELISA. Solid circles, G1; solid squares, G2; solid triangles, G4. *P < 0.001

Antibody response against *H. polygyrus* was slow to develop and on day 35 pi showed a significant IgG_1_ serum response against *Heligmosomoides* ASE in the groups infected with the helminth (Fig. 6a) (*F*_(3, 24)_ = 37.939, *P* < 0.0001]. The response was higher in the group co-infected (G1: 0.362 ± 0.098) than in the mice with monospecific infection (G2: 0.311 ± 0.054) although differences were not significant. No specific IgG_2a_ response to *Heligmosomoides* ASE was observed except for an outlier value (Fig. 6b). Monospecific *Leishmania* infection (G3) elicited a significant IgG apparently related to the IgG_2a_ response (35 days pi) against homologous antigen extract (SLA) whereas no IgG_1_ specific response was observed (not shown).

## Discussion

Under natural conditions, co-infections are the rule in all host species, including humans, [3] and they have a significant impact on disease course outcome, diagnosis and preventive measures [6]. Helminth-protist interactions are very complex ranging from a lack of interference [14] to strong impairment of protective immunity [13]. For the most part, some type of modulation/switching of immune response has been found in the co-infections so far explored. Direct comparison of the results is extremely difficult since the mechanisms involved and final outcome depend on the parasite species and also the schedule of infections (e.g. timing and doses administered). In our experiments, overinfection with *L. infantum* of previously infected mice with *H. polygyrus* strongly impaired the immune response against helminth infection. This interference was evidenced by a worsening of major parameters such as the apparent higher fertility of female worms in early patency and higher helminth burdens at the end of the experiment.

The number of helminths found at the beginning of the patency evidenced a high establishment rate (*c.*40%), in-line with previous observations in primary infections by *H. polygyrus* [24, 25]. It has been reported that a previous infection with *Toxoplasma gondii* led to a higher fecundity of *H. polygyrus* female worms 14 days pi [26]. These results were comparable to those obtained by us with an unrelated protist species, *L. infantum*. Thus, a notable increase in fecal egg output was found in co-infected mice from day 15 to 21 pi without helminth number differences compared to the animals subjected to a single *H. polygyrus* infection (14 days pi). Moreover, we found at the end of the experiment (35 days pi) a significantly higher number of worms in co-infected mice. These findings are consistent with the partial abrogation of the Th2 response in *H. polygyrus* infections [10] and it has also been described in other co-infections with this helminth species [16, 26] irrespective of the experimental design. In *H. polygyrus*/*Trypanosoma congolense* co-infections, no differences were found in female helminth fertility [24, 27]. However, this experiment was not designed to determine the response in primary helminth infections but the acquired resistance after challenge. Our results suggest that the timing, order and protist species involved is apparently less relevant and in both cases immune response polarizes towards a Th1 type this resulting in both higher fecundity and longevity of helminths. The actual mechanistic basis of this interference is poorly known and deserves attention since it is much relevant within the framework of naturally infected hosts where the polyparasitism is the rule [5].

Single infection with *L. infantum* did not elicit significant changes in spleen weight in any of the experimental time points (1 week pi, 4 weeks pi). Intravenous infection of susceptible mice (e.g. BALB/c) with visceral *Leishmania* leads to self-limiting liver invasion and a progressive spleen parasitism. Thus, our results could be possibly related to the pi time elapsed [28]. Mice infected with *H. polygyrus* alone or co-infected with *L. infantum* showed a notable increase in spleen weight, this being consistent with the immune response mounted against the nematode infection. It is noteworthy to indicate that co-infection with *L. infantum* actually caused an early reduction of spleen weights (14 days pi) compared to those seen in the mice with the helminth infection alone. This result supports the interference of the co-infection and the impairment of the characteristic Th2 protective response against the nematode [8, 29]. Poor anti-*H. polygyrus* specific lymphoproliferation in both lymph nodes and spleen was consistent with the unspecific immune suppression (ConA) elicited by nematode infection, particularly in the first parasitic stages (< 14 days pi). Conversely, the immune suppression in *L. infantum-*infected mice (G3) was seen in later stages (day 35 pi), perhaps related to the course of the infection [28]. Visceral *Leishmania* infections cause a mixed Th1/Th2 response [19–21] and *H. polygyrus* is highly effective in blocking the protective Th2 immunity [10]. In our experiment with primary helminth infections, high IgG_1_ anti-*H. polygyrus* levels were found after 35 days whereas no IgG_2a_ was detected. This suggests a Th2 polarized response [29] and, in our experiment, *L. infantum* overinfection actually increased the antibody response against the helminth. A lack of correlation between IgG_1_ levels and protection (e.g. reduced parasite burden or fecal egg output) points towards the limited protective role of the antibodies detected by ELISA [30]. Although not strictly comparable to our experimental approach, it has been observed that human patients co-infected with visceral *Leishmania* and helminths displayed no alteration of the course of leishmaniasis [19] whereas the clinical outcome of cutaneous infection by *L. braziliensis* was significantly influenced by helminth co-infection [17]. It is possible that the initial clinical condition of patients, chemotherapeutic regimen, differential immune response against visceral and cutaneous *Leishmania* and the timing of infections (protist, helminth) could account for the variable response observed.

## Conclusions

Considering all results together, our experiment confirmed the unspecific and specific immune suppression elicited by primary infection by *H. polygyrus* in mice. More importantly, overinfection with *L. infantum* of previously infected animals with the helminth aggravated the suppression, leading to higher parasite burdens, fecal egg output and longevity. Co-infection apparently polarized the immune response towards a non-protective Th1 type. However, no complete Th1/Th2 switch was found since co-infected mice developed a notable specific IgG_1_ response against *Heligmosomoides*. A lack of correlation between protection and IgG_1_ levels suggests the limited role played by antibodies in coping with helminth infection.

## Abbreviations

PBS: phosphate-buffered saline
ELISA: enzyme-linked immunosorbent assay
EPG: eggs per gram
AUC: area under the curve
pi: post-infection
ASE: adult soluble extract
SLA: soluble leishmanial antigen
MTT: 3-(4,5-dimethylthiazol-2-yl)-2,5-diphenyltetrazolium bromide
EDTA: ethylenediaminetetraacetic acid
MLN: mesenteric lymph nodes
SD: standard deviation
SE: standard error
HSD: honestly significant difference
